# Heterogeneous Expression of *Drosophila* Gustatory Receptors in Enteroendocrine Cells

**DOI:** 10.1371/journal.pone.0029022

**Published:** 2011-12-14

**Authors:** Jeong-Ho Park, Jae Young Kwon

**Affiliations:** Department of Biological Sciences, Sungkyunkwan University, Suwon, Gyeonggi-do, Korea; AgroParisTech, France

## Abstract

The gastrointestinal tract is emerging as a major site of chemosensation in mammalian studies. Enteroendocrine cells are chemosensory cells in the gut which produce regulatory peptides in response to luminal contents to regulate gut physiology, food intake, and glucose homeostasis, among other possible functions. Increasing evidence shows that mammalian taste receptors and taste signaling molecules are expressed in enteroendocrine cells in the gut. Invertebrate models such as *Drosophila* can provide a simple and genetically tractable system to study the chemosensory functions of enteroendocrine cells *in vivo*. To establish *Drosophila* enteroendocrine cells as a model for studying gut chemosensation, we used the *GAL4/UAS* system to examine the expression of all 68 Gustatory receptors (Grs) in the intestine. We find that 12 *Gr-GAL4* drivers label subsets of enteroendocrine cells in the midgut, and examine colocalization of these drivers with the regulatory peptides neuropeptide F (NPF), locustatachykinin (LTK), and diuretic hormone 31 (DH31). RT-PCR analysis provides additional evidence for the presence of *Gr* transcripts in the gut. Our results suggest that the *Drosophila* Grs have chemosensory roles in the intestine to regulate physiological functions such as food uptake, nutrient absorption, or sugar homeostasis.

## Introduction

Taste sensing is essential for the survival of all animals, to identify nutrient-rich food sources and avoid harmful substances. Taste, or gustatory, receptors expressed in taste cells recognize distinct non-volatile chemical cues including sugars, amino acids, or bitter compounds. In mammals, G-protein coupled receptors (GPCRs) in the T1R and T2R family mediate the detection of sweet, umami, and bitter taste in the oral epithelium [1]. The T1R family has three distinct subunits that mediate detection of sweet taste (T1R2 + T1R3) or umami and other amino acids (T1R1 + T1R3) as heterodimers. The T2R family encodes over 30 genes encoding different receptors that mediate bitter taste [1]. In *Drosophila*, the *Gustatory receptor* (*Gr*) gene family is composed of 60 members that encode 68 seven-transmembrane receptors through alternative splicing [2], [3], [4], [5]. *Gr5a*, *Gr64a*, and *Gr64f* encode sugar receptors and are members of a subfamily of eight *Gr*s [6], [7], [8]. *Gr66a*, *Gr33a*, *Gr93a*, and *Gr32a* mutants are defective in detecting bitter taste [9], [10], [11], [12]. In recent comprehensive studies of Gr expression in the labellum [13] and larval taste system [14], most Grs appear to be expressed in bitter sensing neurons. Not all Grs are involved in detecting taste; for example, Gr21a and Gr63a are responsible for the CO_2_ response [15], [16].

Recent studies in mammals have established that taste receptors found in the oral epithelium are also expressed in intestinal enteroendocrine cells [17], [18], [19], [20]. Enteroendocrine cells are chemosensory cells in the gut which produce regulatory peptides upon detection of luminal nutrients or chemicals [21], [22], [23]. These regulatory peptides can then induce functional responses by acting on nearby cells or neurons innervating the gut in a paracrine manner, or by acting on distant targets such as the brain in an endocrine manner. In mammals, the GPCR taste receptors and downstream signaling elements including the taste specific G-protein α-gustducin were observed to express in enteroendocrine cells in human and rodent intestines and enteroendocrine cell lines [17], [24], [25], [26]. T1R functions in gut enteroendocrine cells are mainly being explored in relation to glucose sensing, which can lead to systemic effects on glucose homeostasis, appetite, and insulin secretion [22], [23], [27]. T2Rs were shown to be functional in enteroendocrine STC-1 cells, since application of T2R ligands to STC-1 cells induced Ca^2+^ signaling and release of the cholecystokinin (CCK) peptide hormone [28]. However, the functional significance of T2R activation in the intestine is still unclear.

**Figure 1:**
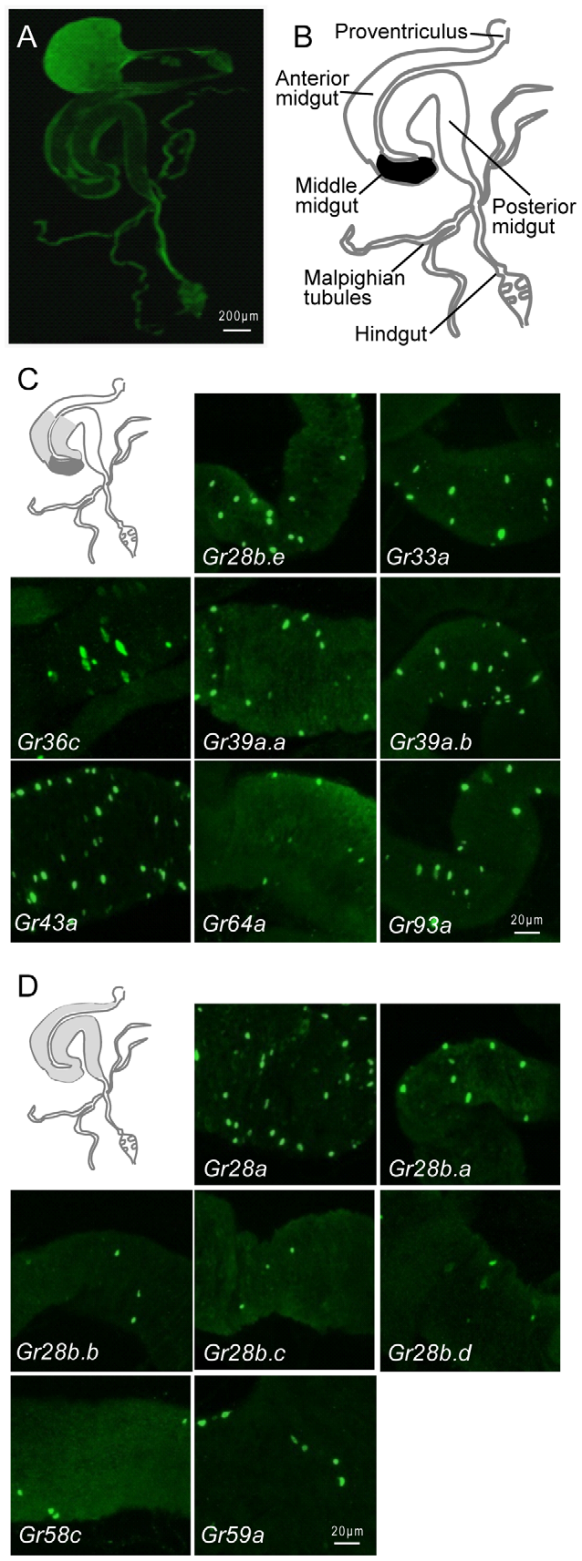
*Gr-GAL4* drivers expressed in the intestine. (A) The *Drosophila* digestive tract visualized by autofluorescence. The crop is highly autofluorescent. (B) A schematic of the digestive tract based on (A). The midgut consists of three regions: the anterior, middle, and posterior midgut. The middle midgut is colored in black. (C) Class I *Gr-GAL4* drivers label cells concentrated in the middle midgut, with expression fewer cells in regions of the anterior and posterior midgut proximal to the middle midgut. (D) Class II *Gr-GAL4* drivers show uniform expression in cells over the entire midgut. Anterior is to the left, posterior to right. Scale bars are as indicated for each panel. *mCD8-GFP* was used as a reporter for the *GAL4* drivers. *Gr-GAL4* driver expression was detected by staining with anti-GFP antibody in this and all subsequent figures.

The *Drosophila* digestive system has many similarities to the vertebrate system in its cell types, development, and genetic control [29], [30], [31]. The *Drosophila* gut is divided into the foregut, midgut, and hindgut, with most nutrient absorption occurring in the midgut, and water and ion homeostasis occurring in the hindgut. The midgut can be divided again into the anterior, middle, and posterior midgut (Fig. 1B) [32]. The middle midgut is characterized by the presence of copper cells which secrete acid to maintain a low pH [33]. The intestinal epithelial monolayer of the *Drosophila* midgut is mainly composed of enterocytes involved in nutrient absorption, interspersed with enteroendocrine cells [30]. Intestinal stem cells dispersed along the basement membrane of the midgut continuously replenish the intestinal cells [30], [34].

Most functional studies of mammalian gut chemosensation have been carried out *in vitro* or by using transgenic mice, and thus it is unclear whether the results accurately reflect the *in vivo* functions of enteroendocrine cells [23]. In this respect, the *Drosophila* gut, with its similarities to the vertebrate gut while being a simpler system, may provide an ideal model to systematically study the *in vivo* chemosensory functions of enteroendocrine cells. In this study, as the first step to establish *Drosophila* enteroendocrine cells as a model system to study chemosensation in the gut, we examine whether enteroendocrine cells in the midgut express Gustatory receptors (Grs) using the *GAL4/UAS* system. RT-PCR analysis provides additional evidence for the presence of *Gr* transcripts in the gut. We identify 12 *Gr-GAL4* drivers which label midgut enteroendocrine cells, and also examine the colocalization of these cells with the regulatory peptides neuropeptide F (NPF), locustatachykinin (LTK), and diuretic hormone 31 (DH31).

## Materials and Methods

### *Drosophila* stocks and transgenic flies

Flies were grown on standard cornmeal/agar culture medium at an average culture temperature of 23°C. All 67 *Gr-GAL4* transgenes used in this study were previously described [13]. *w^1118^* was used as the background to generate transgenic lines [13]. 67 drivers were used to assess expression of the 68 Gr proteins; *Gr23a-GAL4* represents two alternative transcripts of *Gr23a* which share the same 5′ upstream region. Representative lines were selected based on previous studies (A.D., J.Y.K., L.W., F. L., J-H.P., and J.R.C., unpublished results) [13], [14]. At least two independent lines were examined for most *Gr-GAL4* transgenes, with the exception of *Gr10b*, *Gr21a*, *Gr22c*, *Gr22d*, *Gr28b.d*, *Gr28b.e*, *Gr39a.a*, *Gr39a.c*, *Gr39a.d*, *Gr47a*, *Gr59b*, *Gr64b, Gr64c* and *Gr98a*, for which only single lines were available. *UAS-mCD8-GFP* was used as the GFP reporter [35]. *mCD8-GFP* is a membrane marker which allows visualization of entire cell shapes.

### Dissection, antibody staining, and imaging

2- or 3-day-old flies were dissected to examine reporter expression, and males and females were examined separately.

To examine expression in the intestine, whole abdomens were first stained, and the stained intestines were dissected out of the abdominal cavity while mounting.

For antibody staining of whole adult abdomens, dissected abdomens were fixed for at least 2 hours on ice in 4% paraformaldehyde dissolved in phosphate buffered saline (PBS-T, pH 7.2) containing 0.2% Triton X-100. After 3 washes of 20 minutes each in PBS-T, samples were blocked for 2 hours in PBS-T containing 3% normal goat serum. Abdomens were incubated overnight at 4°C with the primary antibody diluted in blocking solution. Washes in PBS-T (3x 20 minutes) were followed by incubation for over 7 hours with the secondary antibody diluted in blocking solution. Samples were rinsed 3x 20 minutes in PBS-T and mounted in 50% glycerol in PBS-T. 4′,6-diamidino-2-phenylindole (DAPI, Sigma) was added to the last wash at a final concentration of 0.5 µg/ml. Unless otherwise noted, all steps were carried out at room temperature.

The primary antibodies used were rabbit anti-GFP (1∶1000) (Molecular Probes); anti-Prospero (1∶10) (Developmental Studies Hybridoma Bank at the University of Iowa); anti-dNPF (1∶1000) (Dr. M. R. Brown, University of Georgia, USA); anti-LTK, anti-DH31 (1∶1000) (Dr. J. A. Veenstra, Universite de Bordeaux, France). The anti-dNPF antiserum was preincubated with FMRF peptides (25 µg/ml, Sigma P4898) at 4°C overnight before use. The secondary antibodies used were goat anti-mouse and goat anti-rabbit IgG conjugated to either Alexa 568 or Alexa 488 (1∶1000) (Molecular Probes).

To locate the acid-secreting middle midgut cells, bromophenol blue staining was done as described [36].

The number of GFP-positive midgut cells for individual *Gr-GAL4* drivers were estimated on a Leica MZ16FA or Leica DM2500 fluorescent microscope. No difference in cell numbers was observed when the same samples were counted under either microscope. We note that an accurate count of GFP-positive cells is difficult to obtain, since GFP intensity differs from cell to cell, and the number of GFP-positive cells can differ depending on magnification conditions.

All images were collected on a Zeiss LSM 510 laser-scanning confocal microscope.

### RT-PCR amplification of *Gr* transcripts

More than 80 flies were dissected to collect intestines, and gut total RNA was extracted using the RNAiso Plus kit (Takara). The RNeasy kit (Qiagen, catalog no. 74104) was used to clean the extracted total RNA. Reverse transcription was performed using the PrimeScript 1^st^ strand cDNA Synthesis kit (Takara), and PCR was performed for 35 cycles using genomic DNA or the synthesized cDNA as templates. To distinguish between genomic and cDNA PCR products, each primer set was designed to span at least one intron. Primers and expected genomic and cDNA PCR product sizes are listed in Table S1.

## Results

### *Gr-GAL4* drivers label cells in the midgut

To systematically examine the expression of the entire family of Grs in the midgut, we used the *GAL4/UAS* system. Gr expression patterns have been successfully analyzed using the *GAL4/UAS* system [3], [5], [12], [13], [14], [37], [38], [39], [40], whereas *in situ* hybridization has been mostly unsuccessful, likely due to low expression levels [2], [3], [5], [6], [12]. Analysis of *Gr-GAL4* driver expression in the adult labellum corresponded well with functional analysis, validating this approach [13]. The expression patterns of *Gr-GAL4* drivers have been extensively studied in adult and larval tissues [13], [14], [39], and each *Gr-GAL4* line was observed to have specific patterns of expression in various tissues including the labellum, multidendritic neurons, and reproductive organs.

A total of 67 *Gr-GAL4* drivers which represent the 68 Gustatory receptors (*Gr23a-GAL4* represents two alternatively spliced forms of *Gr23a* which share a common 5′ region) were examined for expression in the midgut. Representative lines previously observed to have highly penetrant expression levels and patterns were examined (A.D., J.Y.K., L.W., F. L., J-H.P., and J.R.C., unpublished results) [13], [14]. At least two independent lines were examined for most *Gr-GAL4* transgenes, with the exception of *Gr10b*, *Gr21a*, *Gr22c*, *Gr22d*, *Gr28b.d*, *Gr28b.e*, *Gr39a.a*, *Gr39a.c*, *Gr39a.d*, *Gr47a*, *Gr59b*, *Gr64b*, *Gr64c*, and *Gr98a* for which only single lines were available. We examined 2- or 3-day-old male and female adults that contained two copies of both the *Gr-GAL4* driver and *UAS-mCD8-GFP* reporter, and examined more than ten animals for each independent line. Sexual dimorphism was not observed for any of the 67 *Gr-GAL* drivers examined (data not shown). *w^1118^* and *w^1118^; UAS-mCD8-GFP; UAS-mCD8-GFP* animals were stained as negative controls. No GFP expression was observed in *w^1118^* animals and non-specific weak GFP expression was observed in the anterior midgut of *w^1118^; UAS-mCD8-GFP; UAS-mCD8-GFP* animals (data not shown).

**Figure 2:**
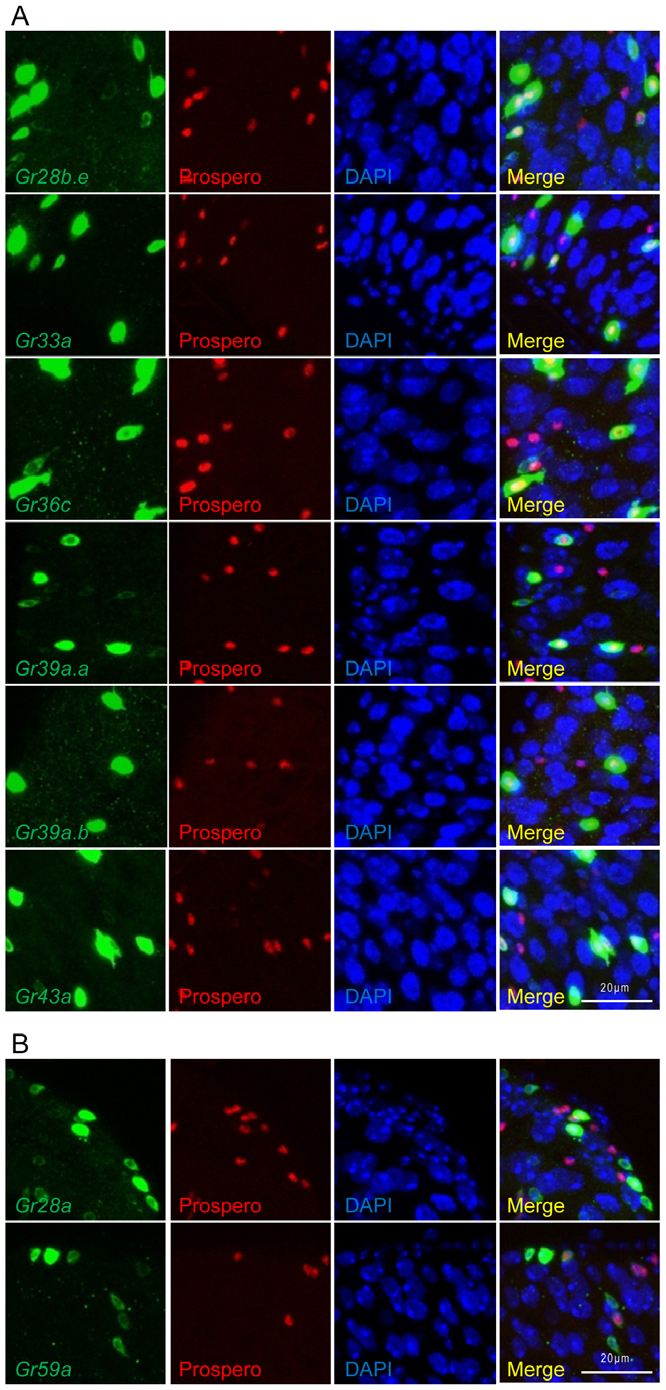
Examples of *Gr-GAL4* drivers expressing in midgut enteroendocrine cells. (A) 6 class I *Gr-GAL4* drivers which label cells that mostly overlap with anti-Prospero-labeled cells (>70%). Prospero is an enteroendocrine cell marker. (B) 2 class II *Gr-GAL4* drivers which label cells of which only a small subset overlaps with anti-Prospero positive cells (<20%). *mCD8-GFP*, which was used as a reporter for the *GAL4* drivers, is a membrane marker which allows visualization of entire cell shapes. Prospero is a transcription factor and is thus nuclearly localized. DAPI staining allows identification of enterocytes, which have large polyploid nuclei.

Of the 67 *Gr-GAL4* drivers, 15 *Gr-GAL4* drivers were observed to label small midgut cells that did not appear to be enterocytes based on DAPI staining (Table 1, Fig. 1 and 2, Fig. S1). Enterocytes can be distinguished through DAPI staining by their polyploid nuclei [30], [34], [41]. We divided the 15 *Gr-GAL4* drivers into two classes based on expression patterns in the midgut. Class I *Gr-GAL4* drivers show expression in cells concentrated in the middle midgut, with expression in fewer cells in regions of the anterior and posterior midgut proximal to the middle midgut (Fig. 1C). Class II *Gr-GAL4* drivers show relatively uniform expression in cells over the entire midgut (Fig. 1D).

**Table 1:** Coexpression of *Gr-GAL4* drivers with markers in the intestine.

class	*Gr-GAL4*	Prospero	NPF	DH31	lines^a^
I	*Gr28b.e*	++	++	−	1/1^b^
	*Gr33a*	++	++	−	1/2
	*Gr36c*	++	++	+	2/2
	*Gr39a.a*	++	++	−	1/1
	*Gr39a.b*	++	++	−	1/3
	*Gr43a*	++	++	−	2/2
	*Gr64a*	++	++	−	1/2
	*Gr93a*	++	++	−	1/1
II	*Gr28a*	+	+	−	3/3^c^
	*Gr28b.a*	++	++	++	2/2
	*Gr28b.b*	−	−	−	2/2
	*Gr28b.c*	−	−	−	2/2
	*Gr28b.d*	−	−	−	1/1^c^
	*Gr58c*	+	−	−	2/2
	*Gr59a*	+	+	−	2/2

++, >70% colocalization with cells labeled by the *Gr-GAL4* driver. +, <20% colocalization with cells labeled by the *Gr-GAL4* driver. −, no colocalization with cells labeled by the *Gr-GAL4* driver.

a Number of lines showing observed expression/number of independent lines analyzed.

b Includes a line obtained from K. Scott.

c Includes a line obtained from H. Amrein.

When the numbers of GFP-positive cells for each *Gr-GAL4* driver were estimated under consistent microscope and magnification conditions (see Materials and Methods), the 8 *Gr-GAL4* drivers that fall into class I are expressed in a wide range of different numbers of midgut cells, ranging from several cells for *Gr93a-GAL4* to as many as 50 cells for *Gr43a-GAL4*. This wide range of numbers suggests that subsets of midgut cells express different combinations of Grs, although definitive conclusions cannot be made without double labeling experiments using different colored markers for each *Gr*. Among the 7 *Gr-GAL4* drivers that fall into class II, *Gr28a-GAL4* (>100 cells) and *Gr59a-GAL4* (≤40 cells) are expressed in large numbers of cells over the entire midgut, while the other drivers are expressed in much fewer cells (≤13 cells). Despite the low numbers of GFP-positive cells, expression was consistent in at least two independent lines for each of these *Gr-GAL4* drivers, with the exception of *Gr28b.d* for which only one independent line was available (Table 1).

Transcripts of 14 of the 15 class I or class II Grs were detected in the intestine by RT-PCR using gut total RNA (Fig. S2 and Table S1). The cDNA product for Gr28b.e was not amplified by RT-PCR.

### 12 *Gr-GAL4* drivers are expressed in midgut enteroendocrine cells

Recent work has provided convincing evidence that enteroendocrine cells have chemosensory functions in the mammalian gut, including studies which show expression of G-protein coupled taste receptors or other taste-signaling proteins in enteroendocrine cells [23], [27]. To examine whether the midgut-expressing *Gr-GAL4* drivers label enteroendocrine cells, anti-Prospero antibody was used as a marker. Prospero is a transcription factor expressed in enteroendocrine cells [30]. 12 *Gr-GAL4* drivers labeled midgut cells that colocalized with Prospero-positive cells, indicating expression in enteroendocrine cells (Table 1, Fig. 2, Fig. S1). All class I *Gr-GAL4* drivers and 4 class II *Gr-GAL4* drivers colocalized with Prospero. Among the *Gr-GAL4* drivers of the four alternative spliceforms of *Gr28b* that fall into class II, only *Gr28b.a-GAL4* was observed to colocalize with Prospero, suggesting that different isoforms of Gr28b have discrete functions in distinct cell types. All class I *Gr-GAL4* drivers and class II *Gr28b.a-GAL4* showed extensive colocalization with Prospero (Table 1, Fig. 2A, Fig. S1A), while only a small subset of cells labeled by the class II *Gr28a-*, *Gr58c-*, and *Gr59a-GAL4* drivers colocalized with Prospero (Table 1, Fig. 2B, Fig. S1B).

### *Gr-GAL4* driver colocalization with cells expressing regulatory peptides

At least 6 different regulatory peptides are known to express in the adult *Drosophila* midgut [36]. Among these, we verified that all neuropeptide F (NPF)-, locustatachykinin (LTK)-, and diuretic hormone 31 (DH31)-positive cells are also positive for Prospero, and are subsets of Prospero-positive cells (Fig. S3). Prospero-positive cells were previously shown to include allatostatin- and tachykinin-positive cells, as well as cells that were not labeled by allatostatin or tachykinin [30].

**Figure 3:**
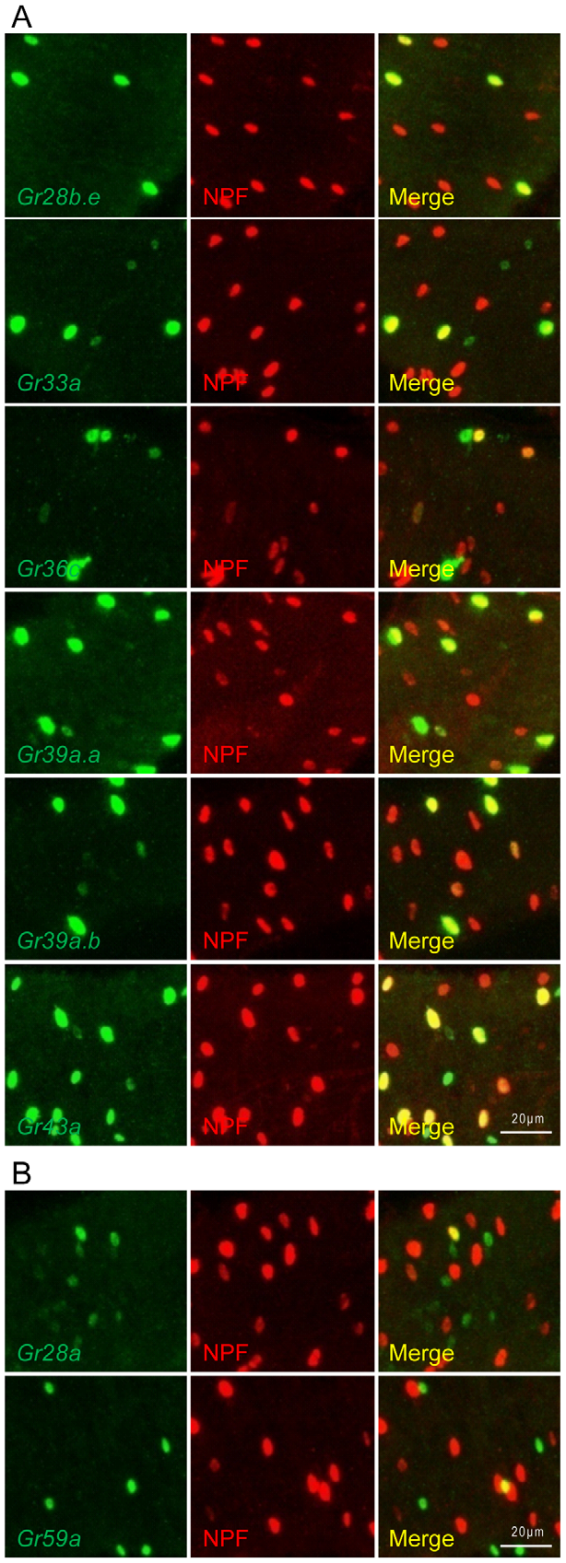
Many midgut enteroendocrine cells labeled by *Gr-GAL4* drivers also express NPF. (A) Examples of *Gr-GAL4* drivers which label midgut enteroendocrine cells of which many, but not all, GFP-positive cells also coexpress NPF, as visualized by staining with anti-NPF antibody. (B) *Gr-GAL4* drivers which drive expression in enteroendocrine cells of which only a few colocalize with NPF-expressing cells.

All class I *Gr-GAL4* drivers labeled midgut cells that mostly colocalized with NPF-positive cells (Table 1, Fig. 3). Among the class II *Gr-GAL4* drivers, only the *Gr28b.a-GAL4* driver labeled midgut cells that mostly colocalize with cells positive for NPF, while only a small subset of the cells labeled by *Gr28a-GAL4* or *Gr59a-GAL4* were also positive for NPF, and *Gr58c-GAL4*-labeled cells did not overlap with NPF-positive cells (Table 1, Fig. 3).

The locustatachykinins (LTKs) are insect tachykinins with homology to the vertebrate tachykinin family which were first described in the locust [42], [43]. LTK antiserum labels enteroendocrine cells all along the length of the midgut [36], as we also observed (data not shown). All NPF-positive enteroendocrine cells were observed to be positive for LTK [36], and correspondingly we also observed that all *Gr-GAL4* drivers that label cells which overlap with NPF-positive cells also label cells that overlap with LTK-positive cells (data not shown).

**Figure 4:**
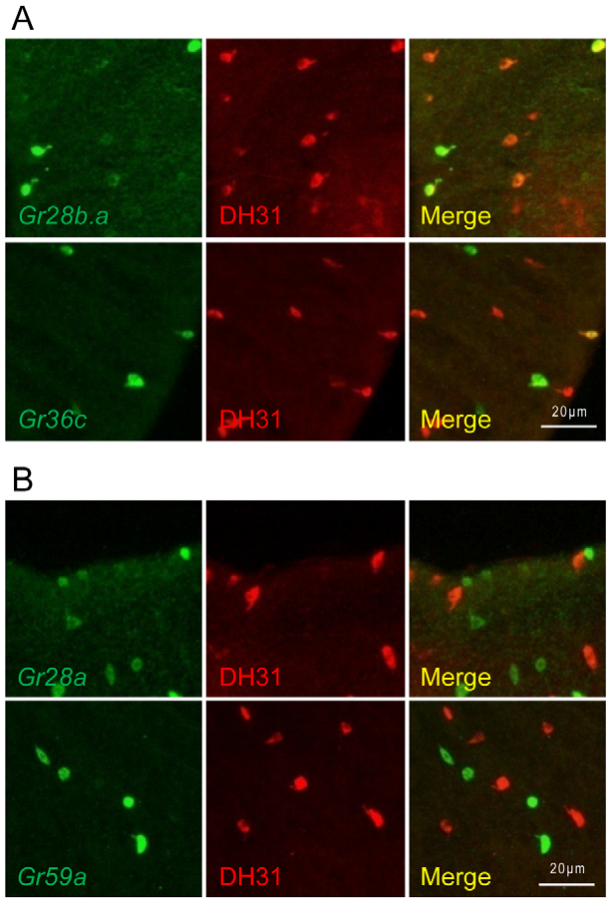
Colocalization of *Gr-GAL4*-labeled enteroendocrine cells and DH31. (A) *Gr28b.a-GAL4* and *Gr36c-GAL4* label enteroendocrine cells which partially colocalize with anti-DH31 stained cells. (B) Examples of *Gr-GAL4* drivers which label enteroendocrine cells that do not colocalize with anti-DH31 stained cells.

Diuretic hormone 31 (DH31) antiserum labels cells in the caudal half of the posterior midgut [36] (data not shown). Most class I *Gr-GAL4* drivers did not express in cells that overlap with DH31-positive cells (Table 1), which agrees with the observation that class I drivers express in cells concentrated around the middle midgut (Fig. 1). Among the class I drivers, only *Gr36c-GAL4* labeled posterior midgut cells which partially overlapped with cells positive for DH31 (Table 1, Fig. 4A). Of the class II *Gr-GAL4* drivers that label enteroendocrine cells, only the *Gr28b.a-GAL4* driver labeled cells that overlapped to a large degree with DH31-positive cells (Table 1, Fig. 4A). *Gr28a-GAL4* and *Gr59a-GAL4* expressed in posterior midgut cells distinct from the DH31-positive cells (Table 1, Fig. 4B).

We also verified that the *Gr28b.b-GAL4*, *Gr28b.c-GAL4*, and *Gr28b.d-GAL4* drivers, which do not label enteroendocrine cells as assessed by lack of colocalization with Prospero (Table 1), do not colocalize with NPF-, LTK-, or DH31-expressing cells (Table 1, data not shown).

## Discussion

Here, we systematically examined the expression of all 68 Grs in the midgut using 67 *Gr-GAL4* drivers. 15 *Gr-GAL4* drivers labeled midgut intestinal cells that do not appear to be enterocytes, and 12 of these show GFP reporter expression in subsets of midgut enteroendocrine cells which express different regulatory peptides. RT-PCR analysis provided additional evidence that the transcripts of 14 Grs are present in the gut. This study is the first to show that *Drosophila* Grs are expressed in midgut enteroendocrine cells, and provides a basis for predicting the roles of the Grs in enteroendocrine cells, which likely include nutrient sensing.

*Gr* expression in the gustatory neurons of the larval and adult taste organs is highly heterogeneous from cell to cell [13], [14]. Consistently, our results also suggest heterogeneity of *Gr* expression in the midgut, where different subsets of midgut cells express different combinations of Grs and regulatory peptides. First, we were able to group the 15 *Gr-GAL4* drivers which label midgut cells that are not enterocytes into two classes based on overall expression patterns in the midgut. Second, colocalization of *Gr-GAL4*-labeled midgut cells with the enteroendocrine cell marker Prospero supported the idea that distinct subsets of midgut cells exist. Although all class I *Gr-GAL4* drivers and class II *Gr28b.a-GAL4* showed extensive colocalization with Prospero and thus appear to be expressed almost exclusively in enteroendocrine cells, the number of GFP-positive cells differs widely from driver to driver. It is not yet clear what cell types the Prospero-negative non-enterocyte cells represent in the class II *Gr28a-GAL4*, *Gr28b.b-GAL4*, *Gr28b.c-GAL4*, *Gr28b.d-GAL4*, *Gr58c-GAL4*, and *Gr59a-GAL4* labeled cells. Third, colocalization of *Gr-GAL4*-labeled midgut cells with regulatory peptides also suggests that different subsets of enteroendocrine cells exist. All class I *Gr-GAL4* drivers showed extensive colocalization with NPF- and LTK-positive cells, and among these only the *Gr36c-GAL4* driver showed some colocalization with DH31-positive cells. The class II *Gr28b.a-GAL4* driver is the only driver observed to show extensive colocalization with NPF-, LTK-, and DH31-positive cells. The class II *Gr28a-GAL4* and *Gr59a-GAL4* drivers only partially overlapped with NPF- and LTK-positive cells. Midgut cells expressing the class II *Gr58c-GAL4* driver did not colocalize with any of the three peptides tested, suggesting that these cells express a different regulatory peptide(s). Double labeling experiments to analyze the coexpression of Grs in subgroups of enteroendocrine cells could provide convincing evidence of heterogeneity of Gr expression in the midgut as well as providing functional clues.

The functions of the NPF, LTK, and DH31 regulatory peptides in the adult *Drosophila* midgut are unknown, although DH31-expressing enteroendocrine cells have been proposed to modulate peristalsis in the junction between the anterior and middle midgut in *Drosophila* larva [44]. It is also unclear if regulatory peptides expressed in the midgut enteroendocrine cells act in a paracrine manner on nearby cells or neurons, or if they act on distant targets such as the brain to influence metabolism or feeding behavior. Analysis of the roles of the heterogeneous populations of enteroendocrine cells suggested by this study may lend insight into the functions of these peptides in conjunction with the Grs.

Many mammalian studies have focused on the role of enteroendocrine cells in glucose sensing [22], [23]. Among the Grs that we found to express in the enteroendocrine cells, *Gr64a* and *Gr43a* stand out as potential sensors of sugars and carbohydrates in the *Drosophila* gut. *Gr64a* is one of the eight members of the sugar receptor subfamily [4]. *Gr64a* is involved in the sensing of various sugars including sucrose and maltose, as well as various di- or trisaccharides or alcohols [6], [7], [8], [45]. In addition, Gr43a and its *Bombyx Mori* ortholog BmGr-9 were found to act as D-fructose-activated cation channels in heterologous expression systems, and BmGr-9 transcripts were observed to express in the *B. Mori* larval gut, suggesting a sensory role in the intestine [46].

The mammalian T2R receptor family mediates bitter sensing in the oral cavity, and the existence of the T2R receptors in the gut and enteroendocrine cell lines has been demonstrated [1], [22], [23]. With the exception of *Gr43a*, *Gr58c*, and *Gr64a*, all of the *Gr-GAL4* drivers we found to label *Drosophila* enteroendocrine cells are expressed in bitter sensing neurons in the adult labellum, which is the fly equivalent of the mammalian tongue [13]. Gr33a is required for sensing many bitter compounds and is essential for responses to aversive taste as well as inhibition of male-to-male courtship [12]. Gr28b is required for the larval avoidance response upon light sensing [47]. Therefore, it may be that the bitter receptors have a general function in mediating aversive responses to undesirable stimuli. Although little is known about the functions of the T2R bitter receptors in the mammalian gut [22], it seems likely that bitter receptors in the gut would sense harmful substances and initiate appropriate protective responses. The *Drosophila* gut may prove to be an ideal simple system to analyze the functions of bitter receptors in enteroendocrine cells. We note that Gr66a, which is coexpressed with Gr33a in the adult labellum and larval taste system [13], [14], is not detected in the intestine. This may be due to limitations of the *GAL4/UAS* system, although *Gr32a-GAL4*, which is broadly expressed in bitter sensing neurons, was also not detected in the intestine.

In summary, we identified Grs expressed in the *Drosophila* midgut enteroendocrine cells. We also observed the colocalization of *Gr-GAL4* drivers with the regulatory peptides NPF, LTK, and DH31. Enteroendocrine cells are difficult to study *in vivo* in mammalian models, despite growing interest in their roles in nutrient sensing and internal regulation [22]. *Drosophila* may prove to be an ideal model to study the roles of heterogeneous populations of enteroendocrine cells in conjunction with the Grs. This study lays the foundation for the molecular and genetic analysis of internal responses that occur upon the sensing of nutrients or harmful substances in the intestine of *Drosophila*.

## Supporting Information

Figure S1 **Additional class I and II** ***Gr-GAL4*** **drivers expressing in midgut cells.** (A) 2 class I *Gr-GAL4* drivers which label cells that mostly overlap with anti-Prospero-labeled cells (>70%). (B) Colocalization of 5 class II *Gr-GAL4* drivers with anti-Prospero positive cells. *Gr28b.a-GAL4* and *Gr58c-GAL4* label cells which overlap with Prospero, and *Gr28b.b-GAL4*, *Gr28b.c-GAL4*, *Gr28b.d-GAL4* label cells that do not overlap with Prospero positive cells. *mCD8-GFP*, which was used as a reporter for the *GAL4* drivers, is a membrane marker which allows visualization of entire cell shapes. Prospero is a transcription factor and is thus nuclearly localized. DAPI staining allows identification of enterocytes, which have large polyploid nuclei.
(TIF)

Figure S2 **Detection of 14 class I or II Gr transcripts in the intestine by RT-PCR.** g, genomic band amplified from genomic DNA; c, cDNA band amplified from cDNA which was reverse transcribed from total RNA extracted from dissected intestines. (A) RT-PCR results of the 15 class I or class II Grs. The cDNA product for Gr28b.e was not amplified by RT-PCR; a band of the same size as the genomic product was amplified. A 6.1 kb genomic band is weakly visible for Gr28b.a. The asterisk marks a non-specific cDNA PCR product for Gr43a. (B) *Gr21a* and *Gr63a* transcripts were assayed as negative controls. (C) *Pros*, *npf*, *sNPF*, and *Dh31* transcripts were assayed as positive controls.
(TIF)

Figure S3 **Neuropeptide F-, locustatachykinin-, and diuretic hormone 31-positive cells are subsets of Prospero-positive cells.** Anti-Prospero, anti-neuropeptide F (NPF), anti- locustatachykinin (LTK), and anti- diuretic hormone 31 (DH31) antibodies were used for immunostaining cells in the midgut. All NPF-, LTK-, and DH31-positive cells are positive for Prospero, and are subsets of Prospero-positive cells.
(TIF)

Table S1 **List of primers used for RT-PCR and sizes of expected PCR products.**
(DOCX)
