# Supplementary figures and images for: Heterogeneous Expression of Drosophila Gustatory Receptors in Enteroendocrine Cells
Source: PLoS One. 2011 Dec 14;6(12):e29022. doi: 10.1371/journal.pone.0029022 (PMC3237578; doi:10.1371/journal.pone.0029022)

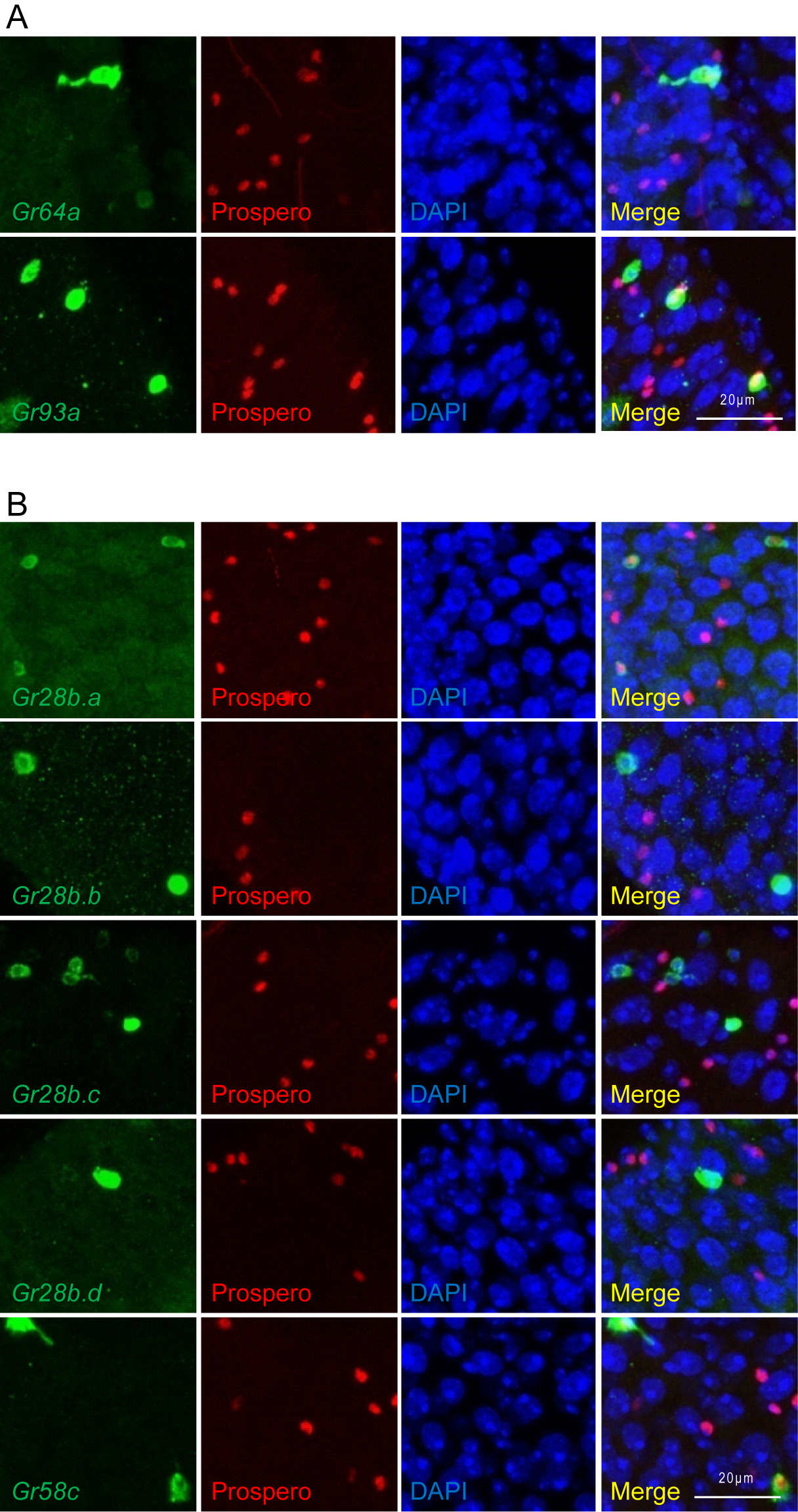

Supplement: Figure S1 — Additional class I and II Gr-GAL4 drivers expressing in midgut cells. (A) 2 class I Gr-GAL4 drivers which label cells that mostly overlap with anti-Prospero-labeled cells (>70%). (B) Colocalization of 5 class II Gr-GAL4 drivers with anti-Prospero positive cells. Gr28b.a-GAL4 and Gr58c-GAL4 label cells which overlap with Prospero, and Gr28b.b-GAL4, Gr28b.c-GAL4, Gr28b.d-GAL4 label cells that do not overlap with Prospero positive cells. mCD8-GFP, which was used as a reporter for the GAL4 drivers, is a membrane marker which allows visualization of entire cell shapes. Prospero is a transcription factor and is thus nuclearly localized. DAPI staining allows identification of enterocytes, which have large polyploid nuclei. (TIF) [file pone.0029022.s001.tif]

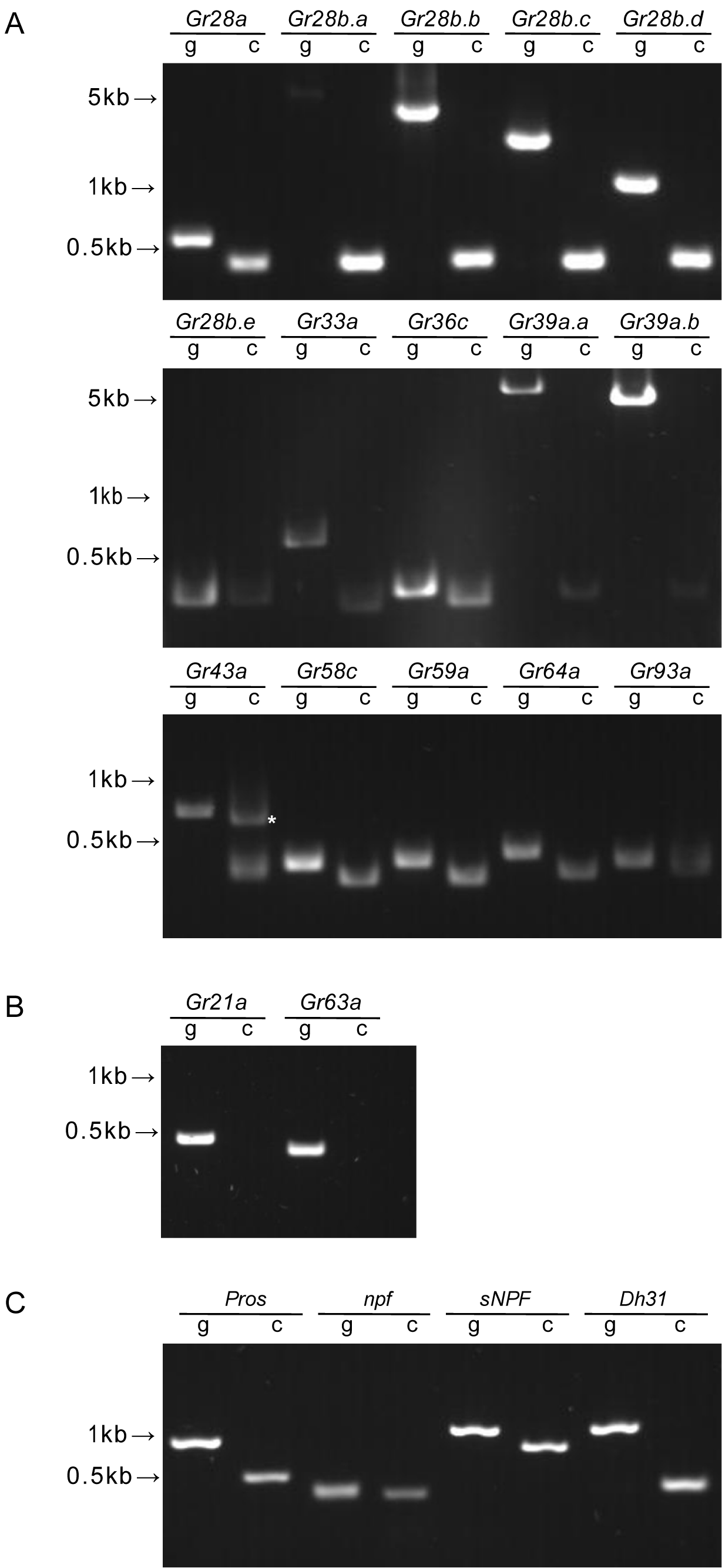

Supplement: Figure S2 — Detection of 14 class I or II Gr transcripts in the intestine by RT-PCR. g, genomic band amplified from genomic DNA; c, cDNA band amplified from cDNA which was reverse transcribed from total RNA extracted from dissected intestines. (A) RT-PCR results of the 15 class I or class II Grs. The cDNA product for Gr28b.e was not amplified by RT-PCR; a band of the same size as the genomic product was amplified. A 6.1 kb genomic band is weakly visible for Gr28b.a. The asterisk marks a non-specific cDNA PCR product for Gr43a. (B) Gr21a and Gr63a transcripts were assayed as negative controls. (C) Pros, npf, sNPF, and Dh31 transcripts were assayed as positive controls. (TIF) [file pone.0029022.s002.tif]

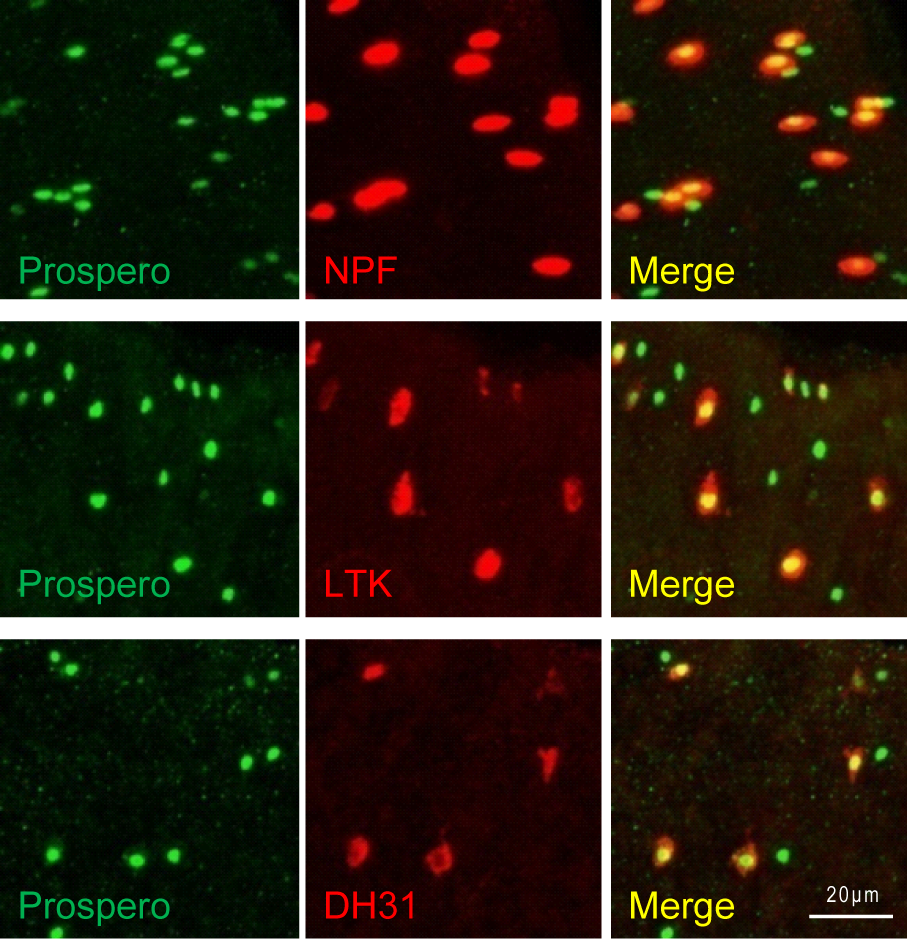

Supplement: Figure S3 — Neuropeptide F-, locustatachykinin-, and diuretic hormone 31-positive cells are subsets of Prospero-positive cells. Anti-Prospero, anti-neuropeptide F (NPF), anti- locustatachykinin (LTK), and anti- diuretic hormone 31 (DH31) antibodies were used for immunostaining cells in the midgut. All NPF-, LTK-, and DH31-positive cells are positive for Prospero, and are subsets of Prospero-positive cells. (TIF) [file pone.0029022.s003.tif]
