# Supplementary material for: Heterogeneous Expression of Drosophila Gustatory Receptors in Enteroendocrine Cells
Source: PLoS One. 2011 Dec 14;6(12):e29022. doi: 10.1371/journal.pone.0029022 (PMC3237578; doi:10.1371/journal.pone.0029022)
Supplement: Table S1 — List of primers used for RT-PCR and sizes of expected PCR products. (DOCX) [file pone.0029022.s004.docx]

**SUPPLEMENTARY TABLES**

**Supplementary Table S1.** List of primers used for RT-PCR and sizes of expected PCR products.

|  | Genomic size (bps) | cDNA size (bps) | Forward primer | Reverse primer |
| --- | --- | --- | --- | --- |
| *Gr28a* | 520 | 303 | 5’-AGCTACAGCCTGCTGGTCAGTG-3’ | 5’-TATGACGGAGGTAACACTGTAC-3’ |
| *Gr28b.a* | 6,120 | 303 | 5’-GGCTTAGTTTGCCTGGCCAAAA-3’ | 5’-ATGTATCTCCATGGACTCCTGT-3’ |
| *Gr28b.b* | 4,476 | 308 | 5’-TTGGAGTTTTCCGGCTGCTCGTC-3’ | 5’- ATGTATCTCCATGGACTCCTGT-3’ |
| *Gr28b.c* | 2,576 | 306 | 5’-GGCACCTTTTCGGTTCTCTATT-3’ | 5’- ATGTATCTCCATGGACTCCTGT-3’ |
| *Gr28b.d* | 1,285 | 308 | 5’-TAAGTTCCAACTGGATGTTGTT-3’ | 5’- ATGTATCTCCATGGACTCCTGT-3’ |
| *Gr28b.e* | 368 | 306 | 5’-AGTTTTCTGATGATCAGTTTGA-3’ | 5’- ATGTATCTCCATGGACTCCTGT-3’ |
| *Gr33a* | 790 | 304 | 5’-ACAGGATTTGGCAATGAGAACA-3’ | 5’-TGGTCTCCAGGAAAATGCCAAAG-3’ |
| *Gr36c* | 369 | 303 | 5’-TGTACGCAATTGCGTTGGATTC-3’ | 5’-CATCGGTGATAGAACCAAAAAT-3’ |
| *Gr39a.a* | 6,012 | 340 | 5’-TTGGTGTCCACCACCACCATCT-3’ | 5’-TTCTATCCAACCCAGTCCCGGT-3’ |
| *Gr39a.b* | 4,742 | 337 | 5’-ATGACTAGCCCCAATCGTCTGC-3’ | 5’- TTCTATCCAACCCAGTCCCGGT -3’ |
| *Gr43a* | 728 | 292 | 5’-AAAGTGCTGGCCCTGGCTCCATAT-3’ | 5’-GTCGATCGTTGAGCTCGAGAGT-3’ |
| *Gr58c* | 329 | 272 | 5’-ACCCATCACGTCGAAATCGTAA-3’ | 5’-CTGGTCATGTTGTGAACGGTT-3’ |
| *Gr59a* | 333 | 272 | 5’-ACCAGGAGCCCTCGCTTGAAAA-3’ | 5’-ACTTGAGTGGACTACGATGGAG-3’ |
| *Gr64a* | 421 | 303 | 5’-CGAAAAGTTGCCCAACTATACCA-3’ | 5’-AGGCGAATGAAGACCACATAGG-3’ |
| *Gr93a* | 362 | 301 | 5’-GTCTTTGTGTCCATTGTGATGG-3’ | 5’-CACCTGTAATGCCGAACTGAAT-3’ |
| *Gr21a* | 490 | 359 | 5’-ATCTCTACCCACTCACCTGGTC-3’ | 5’-CGTTGTGAAGAAGATGACTAGA-3’ |
| *Gr63a* | 361 | 301 | 5’-GAACAGTGCTAATGCCCAGGCAT-3’ | 5’-CGCCAGGAGCACAAAGAAAACCA-3’ |
| *Pros* | 950 | 500 | 5’-TCCGCTCTGGTGGACACCATTG-3’ | 5’-CGCGGCGTGATGCGCGTATCGG-3’ |
| *npf* | 360 | 310 | 5’-ATGTGCCAAACAATGCGTTGCATC-3’ | 5’-TTAGAATATCTCCTCCTCATTAAAA-3’ |
| *sNPF* | 520 | 846 | 5’-ATGTTTCATTTGAAGCGGGAAC-3’ | 5’-TTAGTTCTGTGTCTTTGGTGGG-3’ |
| *Dh31* | 1,169 | 351 | 5’-ATGACAAACCGATGCGCTTGCTT-3’ | 5’-TTAGACATCGGTCTCGGATCGT-3’ |
